# What is the visual behaviour and attentional effort of football players in different positions during a real 11v11 game? A pilot study

**DOI:** 10.12688/f1000research.134231.3

**Published:** 2023-12-01

**Authors:** Charles Ballet, Joana Barreto, Edward Hope, Filipe Casanova

**Affiliations:** 1 Institute Cognitive Neuroscience, University College London, London, England, WC1N 3AZ, UK; 2 Educação Física, Universidade Lusofona de Humanidades e Tecnologias, Lisbon, Lisbon, 376, Portugal; 3 Rehabilitation and Exercise Sciences, University of Essex, Colchester, England, C04 3SQ, UK

**Keywords:** Football, player-role, perceptual-cognitive skills, eye-tracking, decision making

## Abstract

**Background:**

Visual perception has been defined as the first step to a football player’s decision-making process and it plays an important role in performance in sport. The skill of focussing to prioritize relevant cues has been also considered crucial in sport. This pilot study aims to explore the visual behaviour and attentional effort of three football players (mean age 19 ± 0 years old) in specific-role positions; Right-winger (RW), Centre-Midfielder (CM) and Left-Back (LB), in the five seconds before receiving the ball from their teammate.

**Methods:**

Twenty-two male football players performed an 11v11 game, where 24 game sequences (trials) from which 166 fixations were recorded and analysed *via* the Tobii Pro eye-movement registration glasses and software. The gaze behaviour dependent variables were the mean of fixation duration (FD), time to first fixation (TTF), both measured in milliseconds (ms), and the number of fixations (NF) on eight areas of interest (AOIs). AOIs include teammate with and without the ball, opponent without the ball, space around teammate with and without the ball, space around opponent without the ball, ball and undefined. The mean pupil diameter (PD) correlates to the attentional effort and was measured in millimetres (mm).

**Results:**

Descriptive statistics showed nonregular search rate data between the participants in FD, TTF, NF on the AOIs. Mean FD on the ball: (CM, 270 ms), (RW, 570 ms), (CM, 380 ms). They also presented differences in the mean PD during play; (CM: 2.90 mm ± 0.26), (RW: 2.74 mm ± 0.30), (LB 2.77mm ± 0.27).

**Conclusions:**

Albeit the sample size was small, the findings demonstrated a promising way to measure the on-field perceptual-cognitive abilities of football players according to their specific positions, since different playing roles revealed to present distinctive visual and attentional patterns.

## Introduction

Over the last twenty years, perceptual-cognitive skills of athletes in sports have been studied extensively to understand the mechanisms behind anticipation, decision-making, and expertise in sport. ^1^ Perceptual-cognitive skill of an athlete in team sports refers to their ability to use human perception; seeing, hearing and awareness to pick up cues during play. ^1^ Those would be then integrated and processed with existing (tactical) knowledge so that the right sporting decision could be actioned. ^2^ It has been clearly demonstrated that athletes with higher perceptual-cognitive skills have better anticipation and decision-making abilities. ^3^

Research has shown that attention in sport is as important as perception for performance. ^4^ Indeed, visual attention (focus) and visual perception are distinct, yet closely linked. ^4^^,^^5^ From a coaching and professional perspective, visual perception is defined as the first step of football players’ decision-making process. ^6^ A player scans the field during a specific style of play to decide their next action accordingly. ^7^ Visual attention is what enables this player to pick-up all important cues and ignore the less relevant before making their decision. ^4^

When discussing attention, it is important to distinguish between covert and overt attention. ^8^ The covert is characterised by the attention following the movements of the eyes (linked to central vision). ^8^^,^^9^ Overt attention is directed elsewhere than where the eyes are fixating (linked to peripheral vision). ^8^^,^^9^

A football game is characterised by 22 athletes whose movements, speed, body, and positioning, vary continuously. ^10^^,^^11^ As a result, football players would use their visual perception and attention differently to pick-up cues such as spaces, teammates, opponents to intend to make the right decision at the right time. ^9^ As a result, we believe it is important for coaches to be aware of individual players’ visual patterns and attention.

Research on visual perception evidenced that 80% of the information taken from the environment is done through the eyes. ^12^ This allows a player to analyse the game situation and recognised patterns of play. ^12^ Therefore, visual perception is crucial for spatial awareness. ^12^ Visual perception has been previously studied *via* “scanning”, which is the amount of time a player moves their head towards and away from a ball, teammate, or opponent; not considering their gaze behaviour. ^10^^,^^13^ Although, visual exploration can also be done through body movements, it is ultimately through the eyes that the information is mainly picked up and processed. ^10^

When it occurs, the eyes move and fixate at a specific cue, this process is characterised by the steadiness of the gaze at a point of interest. ^14^ It is commonly associated to central vision since it enables individuals to see details clearly and sharply. ^14^ For example, fixation happens when a player keeps their eyes (and therefore gaze) on the ball to get a clearer vision of it. ^12^ Fixation has been previously linked to football performance since its duration can vary depending on the skill level of an athlete and task constraints existing in the sport environments. ^15^ An increase in fixation duration can suggest that the player may have more interest in what is fixating at, and/or processing the information. ^12^ Although there has been a general assumption that the players attention is where they fixate, it has also been evidenced otherwise. ^16^ As an example, a player can look at the most obvious free teammate but pass the ball to another teammate they spotted using their peripheral vision. ^1^

Most visual perception studies explored gaze behaviour following Gibson’s theory of perception-action coupling, which explains the mechanism behind the coordination between what we see and what we do. ^17^ During a football game, players are confronted with ever-changing dynamic situations and constraints which they need to consider constantly before their next actions. For example, a player who is in possession of the ball would have to decide to either shoot at goal, pass to a teammate, or dribble the ball depending on how far the goal is and how his/her teammates and opponents are positioning and moving themselves on the pitch. ^18^

Aksum *et al.* ^10^ conducted an on-field observational study investigating eye movements of midfield players during a 11v11 match play. They analysed visual fixation when the ball was at play, during both defence and attack phases. Moreover, the forementioned authors measured fixation duration and the total time spent (as a percentage) of viewing each fixation’s location, categorising the locations as ball, teammate, opponent, space and other. ^10^ They provided valuable insight from their experiment showing that elite midfield players use longer fixation duration (242.49 ms) when facing several cues such as space, teammates, and opponents. ^10^ Furthermore, midfield players fixate more at the player in possession of the ball during a defensive phase of play than during an attacking phase. ^10^

Although their experiment provided an understanding into the gaze behaviour of midfield players, more research is needed, as it could be argued that football players, including backs and forwards, have different roles on the pitch and therefore might use different gaze strategies. ^19^ Another limitation of their research lays on the fact that they did not explore visual attention.

As previously stated, visual perception and attention are closely linked and integrated within the decision-making process of a football player. ^4^ Therefore, our intention was to investigate visual perception by studying our participants gaze behaviour but also their attention by measuring their pupil size during play.

A laboratory study investigated attentional effort of expert and novice horse riding athletes by measuring pupil diameters using video simulations. ^20^ The results found evidenced that the expert group displayed a higher increase of their pupil size diameter than the novice group. ^20^ Conversely, another study highlighted that a player owning a higher amount of tactical knowledge requires less cognitive effort during a laboratory video test. ^21^ This difference could be pointed because football is a team sports with complex situations compared to horse riding. ^10^^,^^20^

In this observational pilot experiment, we explored the gaze behaviour and attention of three male footballers who played as a left-back (LB), right-winger (RW), and centre-midfielder (CM). The focus was on the five seconds before receiving the ball from a teammate in an 11v11 game. The analysis of the visual (search rate, search order) and attentional data was carried out when the investigated player's team was in possession of the ball.

## Methods

### Participants

Twenty-two male football players (mean age 19 ± 0 years old; 6.67 ± 3.79 years of football practice, which correspond to a total 1334 hours) who play as amateurs in the Portugal National University Championships were recruited. We contacted football coaches of the Lusófona University *via* email who forwarded our invitation letter to the players to voluntary take part to the experiment. They all took part in a 15-minute pre-competitive football game, which were separately recorded the visual behaviour of three of those participants who played as a LB, CM and RW were each analysed and recorded at different moments of an 11 v 11 football game. Participants reported normal or corrected-to-normal levels of visual function. The study complied with the safety guidelines of the Tobi eye tracking devices and was approved by the Ethics Committee of Lusófona University (protocol number M25A21), and the UCL Research Ethics Committee (project identification number 7067/001) which are in accordance with the Declaration of Helsinki. All participants provided voluntary written informed consent, where all procedures were explained in detail, from the data collection to the publication stage.

### Apparatus

The Tobii Pro Glasses 2 ^®^ (Tobii Pro AB, Stockholm, Sweden) eye-movement registration system was worn by each participant during an 11 v11 football pre-competitive match on a full-size pitch. The Tobii Pro Glasses 2 ^®^ is a binocular eye tracker that records the point-of-gaze onto a video image of the scene, measuring the relative position of the pupil and corneal reflection. The image recorded was then analysed *via* the Tobii Pro Lab software (Version X, Tobii Pro AB, Stockholm, Sweden). The Tobi Pro Lab Software was utilized on a Dell Venue 11 Pro 7130, Windows 8/8.1 Pro tablet at a rate of 50 Hz. It is important to highlight that players were not recorded simultaneously. The visual behaviour and attentional effort of three football players were investigated in the five seconds before receiving the ball from their teammate. Jordet *et al.* ^13^ previously investigated the scanning frequency (players looking over their shoulders) per seconds in the last ten seconds of a team possessing the ball. In contrast to Jordet’s preference for a 10-second window and focus on scanning (head movement counts), ^13^ our analysis instead delves into gaze behaviour, specifically eye movements measured in milliseconds. While a more extended timeframe might give additional information, we deliberately opted for a concise duration (5 seconds). This decision considers the balance between data richness and practicality, preventing an overload of information for coaching staff and aligning with the real-time nature of players' actions in a game.

The procedures were carefully explained to the participants before the beginning of the experiment. The eye-tracking glasses were well fitted onto the participant’s face who also worn a vest holding the recording unit in a small pocket between the shoulder blades. To ensure high gaze data quality, calibration procedures were carried out by asking the participants to focus on the center-point of the calibration card held in front of them for five seconds. Each investigated participant practiced for five minutes playing football while wearing the eye tracker to ensure familiarity with the testing protocols. In the experiment, our three investigated participants took part in a 20-min 11v11 pre-competitive football game. Each of them worn the Tobii eye tracker for about 5 minutes.

To control for possible learning biases, no feedback was provided during performance.

### Visual search behaviours

*Search rate*

The measurement of visual search rate comprised those of the number of fixations (NF); characterising how often each player looks at each of the eight areas of interest (AOI), as per Casanova *et al.* ^12^: the ball (B), an opponent without the ball (ONB), the space around opponent without the ball (Space around ONB), any space around a teammate without the ball (space around TNB), space around a teammate with ball (space around TB), teammate with ball (TB), teammate without the ball (TNB) and undefined (U). The “undefined” category is characterised by any gaze data which would not fall into any of the other areas.

The search rate was measured *via* the fixation duration (FD) (in milliseconds; ms) which reveals how long each player looks at each of the eight AOIs.

The gaze data were measured *via* the Tobii Pro lab software *via* metrics analysis and tracking pursuit analysing data frame-by-frame using a sampling rate of 50 Hz. The velocity-threshold identification (IV-T) algorithm was used to classify the different eye movements depending on their velocity, measured in visual degrees per second (°/s). This threshold enables the categorization of the raw gaze data into different eye movements as saccades and fixations. ^22^ For instance, if the velocity is above the threshold, it would be categorised as a saccade. On the contrary, if the velocity turns out to be below the threshold for a minimum duration of 120 ms, the eye movement data would be classified as a fixation. In this experiment the IV-T filter was set up so that a fixation presents with a minimum threshold of 120 ms, with velocity below the threshold of 100 visual degrees per second (°/s). The filter was set up with those values because the subjects, targets and objects would be constantly moving under dynamic situations. ^23^

The analysis of the eye tracking data *via* the Tobi Pro lab software was done *via* assisted mapping of the gaze data point (gaze circle) on to a fixation location and into new coordinate system. In the study conducted by Aksum *et al.* ^10^ the gaze circle was set at 100% so that it could comprise more than one object of interest. For instance, one fixation could include three different areas such as the ball, teammate, and opponent. ^10^ In the present study, we choose to set the gaze circle at 1% to contain only one object of interest, so to make the results more precise.

*Search order*

Search order also defined as fixation order is characterised by the search sequence or order used by the players. ^24^ The search order was measured by analysing the mean time to first fixation (TFF), indicating when each player looks at each AOI. ^25^ A smaller mean time to first fixation value on a specific AOI would indicate that the player looks at this specific AOI earlier in comparison to other AOIs. ^25^

### Attentional effort

*Measure of pupil dilatation*

We measured the size of the pupil of each of three players which is meant to reflect their attentional effort. ^26^ Pupil data often carries “noises” which are data that cannot be interpreted. ^27^ As a result, the moving average noise reduction filter of the Tobii Pro software was used to filter our data. It produces an output data by creating an arithmetic mean of several data points from the input data. ^28^ The moving average filter also makes an average of the right and left eye data, even in the event of only one eye being recorded. ^22^

To demonstrate changes in attentional effort, previous studies measured a baseline (at rest) and a “ *post-stimulus*” mean value of a participant‘s pupil diameter. ^20^ Subsequently, the baseline of the pupil data of each player investigated was obtained by measuring the mean of the pupil diameter during the calibration of the eye tracker. The “ *post-stimulus*” value of the pupil dilation of each player was also measured during the five seconds before receiving the ball, as per the gaze behaviour measurements.

*Reliability*

Test-retest reliability comprised a 20-day interval for re-analysis to avoid any familiarity effects with the task performed using the Cohen’s Kappa test. ^29^ Moreover, reliability was verified through the reassessment of more than 25% of trials, as suggested in the literature. ^3^

### Statistical analysis

The distribution of data sets ^30^ was analysed using Shapiro-Wilk tests. Only descriptive statistics for the results analysis was used since this pilot study had a small sample size and, therefore, is statistically underpowered. ^31^ Descriptive analyses were performed using the Statistical Package for Social Sciences software V24.0 (IBM SPSS Statistics for Mac, Armonk, NY: IBM Corp.) (RRID:SCR_002865).

## Results

### Fixation duration (FD)

**Figure 1.**
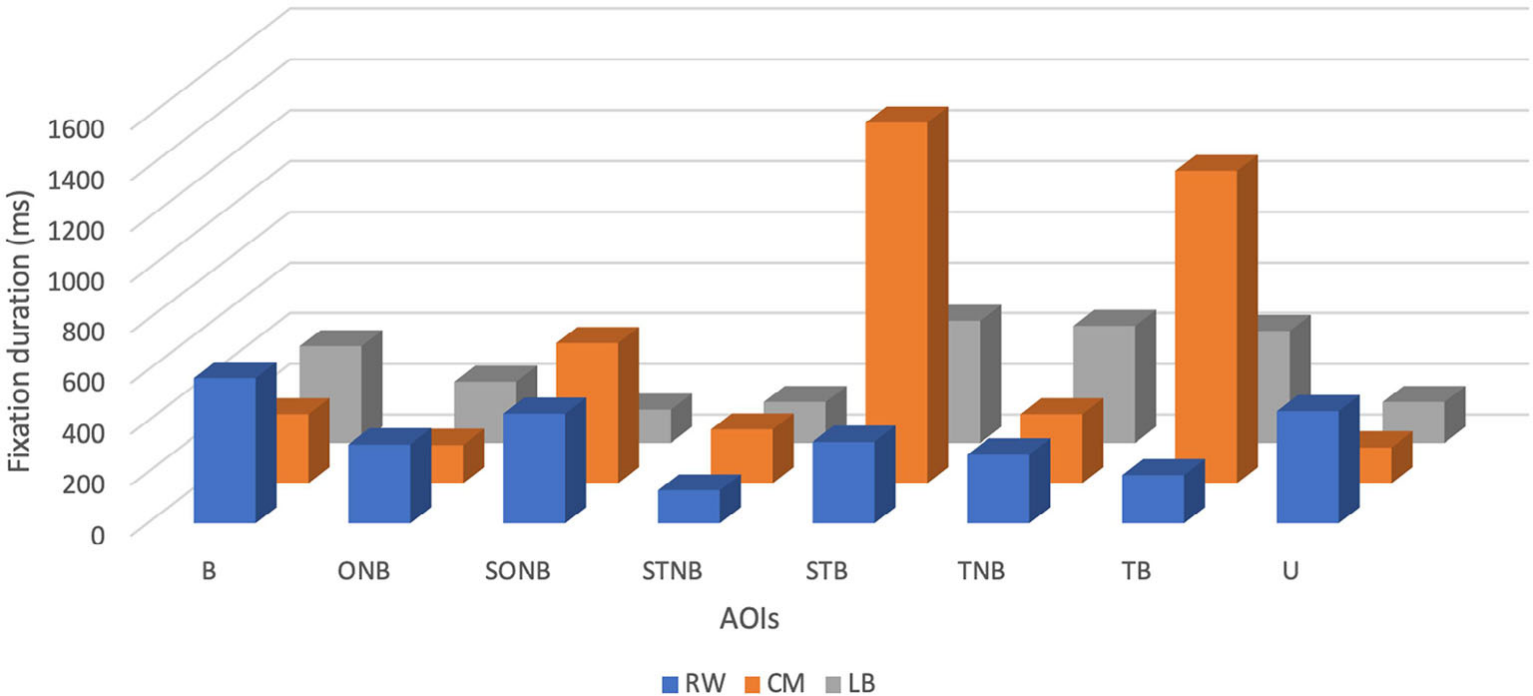
Fixation duration (ms) of players (right winger (RW), centre midfield (CM) and left back (LB)) by their position during the five seconds before receiving the ball.

Descriptive analysis revealed that the mean FD of the CM was the highest and accounted for 530 ± 509.42 ms, with minimum and maximum values being 140 ms and 1420 ms, respectively (see Table 1 and Figure 1). The mean FD of the RW was the second highest at 332.50 ± 143.10 ms, with a minimum value of 130 ms and a maximum value of 570 ms. Finally, the mean FD of the LB was the lowest and accounted for 306.25 ± 149 ms, with a minimum value of 130 ms and a maximum value of 480 ms.

**Table 1.** Mean (m) and standard deviation (±sd) of search rate; fixation duration (ms) and number of fixations obtained in centre-midfielder (CM), right winger (RW) and left back (LB) players.

(Search rate)	Player role					
	Centre midfielder		Right winger		Left back	
	Fixation duration	Number of fixations	Fixation duration	Number of fixations	Fixation duration	Number of fixations
**AOIs**						
(Ball)	270 ± 509.42	25 ± 8.07	570 ± 143.10	19 ± 5.04	380 ± 149	16 ± 4.41
(Opponent No Ball)	150 ± 509.42	2 ± 8.07	310 ± 143.10	6 ± 5.04	240 ± 149	6 ± 4.41
(Space Opponent No Ball)	550 ± 509.42	3 ± 8.07	430 ± 143.10	4 ± 5.04	130 ± 149	7 ± 4.41
(Space Teammate No Ball)	210 ± 509.42	2 ± 8.07	130 ± 143.10	9 ± 5.04	160 ± 149	7 ± 4.41
(Space Teammate Ball)	1420 ± 509.42	6 ± 8.07	320 ± 143.10	7 ± 5.04	480 ± 149	12 ± 4.41
(Teammate No Ball)	270 ± 509.42	2 ± 8.07	270 ± 143.10	6 ± 5.04	460 ± 149	8 ± 4.41
(Teammate Ball)	1230 ± 509.42	2 ± 8.07	190 ± 143.10	5 ± 5.04	440 ± 149	7 ± 4.41
(Undefined)	140 ± 509.42	1 ± 8.07	440 ± 143.10	3 ± 5.04	160 ± 149	1 ± 4.41

The CM participant showed fixations of longer duration on SONB (550 ± 509.42 ms), STB (1420 ± 509.42 ms) and TB (1230 ± 509.42 ms), related to RW: SONB (430 ± 143.10 ms), STB (320 ± 143.10 ms), TB (190 ± 143.10 ms) and LB gaze data: SONB (130 ± 149 ms), STB (480 ± 149 ms), TB (440 ± 149 ms). The LB participant made longer fixations only on the TNB (460 ± 149 ms), related to RW (270 ± 143.10 ms) and CM gaze data (270 ± 509.42 ms).

### Total (TNF) and average number of fixations (NF)

The highest TNF was for LB which accounted for 64, associated with a mean value of 8 ± 4.41, a minimum value of 1 and a maximum of 16.

The second highest TNF was for the RW which was 59, with a mean value of 7.37± 5.04 and minimum and maximum values of 3 and 19, respectively.

We found that the TNF for the CM was 43 and the mean value of 5.36 ± 8.07. The minimum and maximum of NF were 1 and 25, respectively.

For the number of fixations, LB presented with the highest values SONB (7 ± 4.41), STB (12 ± 4.41), TNB (8 ± 4.41) and TB (7 ± 4.41). Whereas RW showed the highest value of numbers of fixations on ONB (6 ± 5.04), STNB (9 ± 5.04), and U (3 ± 5.04) related to the other participants. Lastly, CM only had the highest number of fixations 25 ± 8.07 on B related to RW (19 ± 5.04) and LB (16 ± 4.41).

### Time to first fixation (TFF)

Descriptive statistics showed that across the explored plays the mean TFF of the LB was the highest and accounted for 22770 ± 18441.24 ms, with a minimum value of 620 ms and a maximum value of 53900 ms.

**Figure 2.**
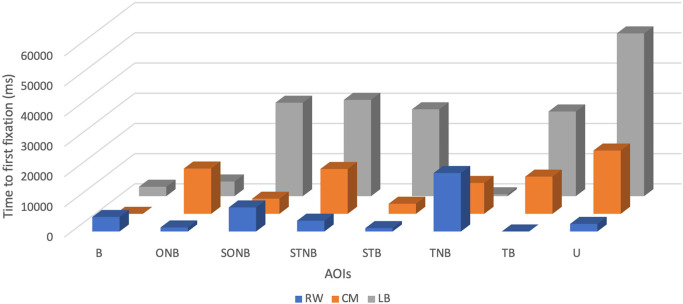
Time to first fixation (ms) of players by their position (right wing (RW), centre midfield (CM) and left back (LB)) during the five seconds before receiving the ball.

The mean of TFF of CM was the second highest and accounted for 10211.25 ± 7000.32 ms, with a minimum value of 0 ms and a maximum value of 20960 ms (see Table 2 and Figure 2).

**Table 2.** Mean (m) and standard deviation (±sd) of search order; time to first fixation (ms) obtained in centre-midfielder (CM), right winger (RW) and left back (LB) players.

(Search order)	Player role		
	Centre midfielder	Right winger	Left back
	Time to first fixation	Time to first fixation	Time to first fixation
**AOIs**			
(Ball)	0 ± 7000.32	4790 ± 6289.68	3120 ± 18441.24
(Opponent No Ball)	15000 ± 7000.32	1330 ± 6289.68	4860 ± 18441.24
(Space Opponent No Ball)	5000 ± 7000.32	7970 ± 6289.68	30950 ± 18441.24
(Space Teammate No Ball)	14850 ± 7000.32	3570 ± 6289.68	31890 ± 18441.24
(Space Teammate Ball)	3340 ± 7000.32	1110 ± 6289.68	28770 ± 18441.24
(Teammate No Ball)	10230 ± 7000.32	19360 ± 6289.68	620 ± 18441.24
(Teammate Ball)	12310 ± 7000.32	0 ± 6289.68	28050 ± 18441.24
(Undefined)	20960 ± 7000.32	2500 ± 6289.68	53900 ± 18441.24

For the time to first fixation, the LB participant showed the highest values for the most AOIs, SONB (30950 ± 18441.24 ms), STNB (31890 ± 18441.24 ms), STB (28770 ± 18441.24 ms), TB (28050 ± 18441.24 ms), U (53900 ± 18441.24 ms) related to RW: SONB (7970 ± 6289.68 ms), STNB (3570 ± 6289.68 ms), STB (1110 ± 6289.68 ms), TB (0 ± 6289.68 ms), U (2500 ± 6289.68 ms) and CM: SONB (5000 ± 7000.32 ms), STNB (14850 ± 7000.32 ms), STB (3340 ± 7000.32 ms), TB (12310 ± 7000.32 ms), U (20960 ± 7000.32 ms). The RW participant displayed the highest values for B (4790 ± 6289.68 ms) and TNB (19360 ± 6289.68 ms), related to CM: B (0 ± 7000.32 ms), TNB (10230 ± 7000.32 ms) and LB: B (3120 ± 18441.24 ms), TNB (620 ± 18441.24 ms). Contrary, the CM participant achieved the highest values only for the ONB (15000 ± 7000.32 ms), related to RW (1330 ± 6289.68 ms) and LB (4860 ± 18441.24 ms).

The mean TFF of the RW was the lowest (5078.75 ± 6289.68 ms) with a minimum value of 0 ms and a maximum value of 19360 ms.

### Pupil diameter

**Figure 3.**
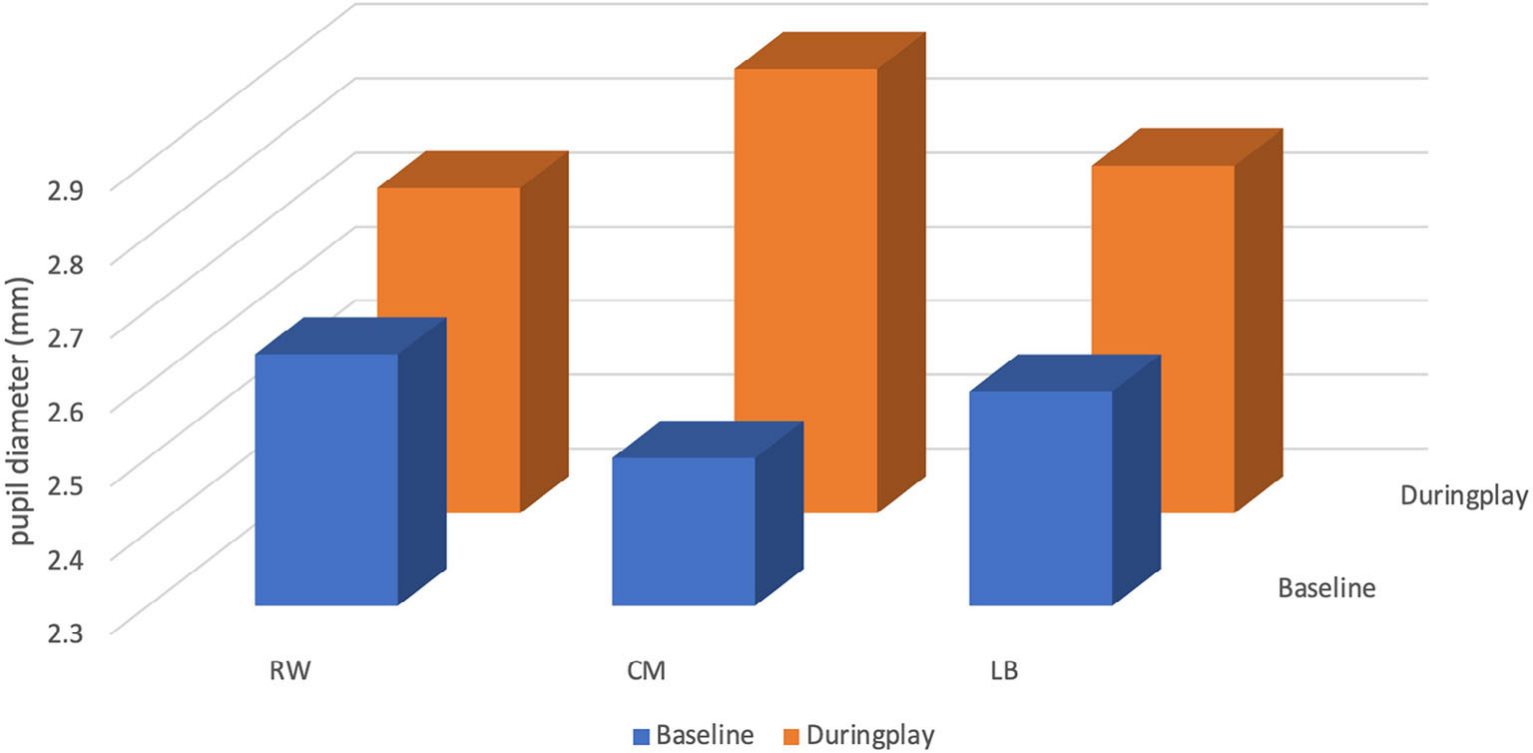
Pupil diameter (mm) of players (right winger (RW), centre midfield (CM) and left back (LB)) by their position during the five seconds before receiving the ball.

The mean pupil size of the CM at baseline was 2.50 ± 0.20 millimetres (mm), with a minimum value of 2.21 mm and a maximum value of 3.34 mm. During play, the CM’s mean pupil size increased by 0.40 mm to 2.90 ± 0.26 mm, with a minimum value of 2.34 mm and a maximum value of 3.63 mm (see Table 3 and Figure 3).

**Table 3.** Mean (m) and standard deviation (±sd) of attentional effort; pupil diameter (mm) obtained in centre-midfielder (CM), right winger (RW) and left back (LB) players.

	Player role		
	Centre midfielder	Right winger	Left back
Attentional effort	Pupil diameter	Pupil diameter	Pupil diameter
Baseline	2.50 ± 0.20	2.64 ± 0.18	2.59 ± 0.35
During play *	2.90 ± 0.26	2.74 ± 0.30	2.77 ± 0.27

* During the five seconds before receiving the ball from a teammate.

The mean pupil size of the RW at baseline was 2.64 ± 0.18 mm, with a minimum value of 2.22 mm and a maximum value of 3.04 mm. During play, the RW’s mean pupil size increased by 0.10 mm to 2.74 ± 0.30 mm, with a minimum value of 2.22 mm and a maximum value of 4.59 mm.

The mean pupil size of the LB at baseline was 2.59 ± 0.35 mm, with a minimum value of 2.38 mm and a maximum value of 5.70 mm. During play, the RW’s mean pupil size increased by 0.18 mm to 2.77 ± 0.27 mm, with a minimum value of 2.35 mm and a maximum value of 3.89 mm.

The CM participant was the footballer who more increased the values for pupil diameter variables from “baseline” to “during play”, related to the RW and LB participants.

## Discussion

This pilot experiment was carried out to explore gaze behaviour and attentional effort of football players from LB, RW and CM during the five seconds before receiving the ball from a teammate on 11v11 game. As expected, in the present study we observed dissimilar gaze behaviour and attentional effort from each football player ( *i.e.*, CM, RW and LB).

### Visual search behaviours

The mean FD of the CM was the highest of all the specific positions analysed. It can be argued that the CM tends to fixate for longer periods of time than the other players, in which is associated with previous studies which explained that the CM players display higher value of mean FD since they usually act as a link between attack and defence. ^19^ Hence, CM players must process more information because they consistently need to scan their surroundings. The RWs, who can be considered as attacking midfielders, displayed the second highest mean value of FD. This contradicts previous research which suggested that attacking players display a lower scanning rate because they usually operate against temporal and spatial in critical areas. ^19^ Likewise, players located at the peripheries have also restricted use of their vision field since they do not need to gather information from outside the side line. ^13^ Lastly, LB presented with the lowest FD mean value due to having proximity to the goals.

Analysis of the mean FD on AOIs showed that CM spent more time looking at spaces, particularly at STB than ONB. This reveals that the CM player had more spatial awareness before receiving the ball, which has been evidenced to enable a player to analyse different options and increase the chance of success of their next tactical action. During the five seconds before receiving a pass, it seemed that the RW was more fixating at the ball than the spaces taking individually; STB, STNB and SONB which might indicate that the RW player tended to do more “ball watching”. Ball watching has been commonly associated with lower league and amateur players’ lack of spatial awareness and technical experience which led them to focus solely on the ball. ^3^^,^^9^ Interestingly, the LB spent most of time fixating at STB and least time on SONB. This could be interpreted that the LB somewhat fixates also at spaces. Nevertheless, it is important to highlight that LB longest mean FD on STB, still remains lower than that of CM and RW’s on that same AOI. This could also be explained by his position on the field, as previously mentioned.

It can be observed that despite CM looking at the ball more times, the player did not spend as much time fixating at it but rather fixated longer at STB. FD and its relation to cognitive process has been deemed as intuitive. ^20^ Nevertheless, it has been conjectured that FD appears to be longer with more a complex task and situation. ^2^ As a result, it can be argued that CM tends to fixate longer at STB because he needed to process more complex information. Moreover, it is important to mention that STB could entail the space surrounding two different teammates who might have possessed the ball during the five seconds before CM received a pass. Contrary to the CM, the RW had the tendency to fixate more often at the ball and longer on the ball, while less fixating at STNB. Indeed, the same pattern could be observed for LB and RW, both of whom fixated at the ball more. ^21^ Those findings could be explained by the fact that our participants are all university level, who are known to do more “ball watching” than their elite counterparts. ^3^^,^^9^

The benefits of using TTF as an outcome measure include communicating the visual pattern order of each player. ^25^ Interpretation of TTF on (U) category was not included because it would provide no relevant information in that specific analysis. Subsequently, it can be put forward that during the five seconds before receiving the ball, the CM tends to look first at the ball, then STB followed by SONB, TNB, TB, STNB, and ONB. RW would look at the TB, STB, ONB, STNB, B, SONB and TNB, in order. The LB presented with a different visual search order characterised by firstly fixating at TNB then B followed by ONB, TB, STB, SONB, and STNB.

### Attentional effort

As far as we are aware, only a few research studies ^20^ in sport have used pupil diameter measurements to provide information on player’s focus. As a result, it may be difficult to debate our findings to other research studies. Nevertheless, the CM showed a higher increase in pupil size diameter by compared to RW and LB, which could be seen regarded as a trait of expertise. Conversely, another study presented that their players with the lowest attentional effort had the highest tactical knowledge. ^21^ For instance, lower and higher tactical knowledge players displayed mean pupil diameter 3.13 mm ± 1.24 and 3.09 mm ± 1.39, respectively during verbalization of their gameplay decision. ^21^ This contradicts our previous statement since CM presented with the highest cognitive effort during play. Those differences between theirs and our results could be explained by the fact that their study was done with academy players from a higher league (Brazilian first division soccer club) watching a video on a screen. ^21^ Aksum *et al.* ^10^ also highlighted discrepancies when comparing their findings to those found in laboratory studies, showing that players investigated in those studies presented on average with longer fixation duration.

Interestingly, a recent study which looked at evaluating mental load (attentional effort) in training sessions, showed that the pupil diameter of soccer players changes depending on the time spent training. ^5^ Although this study had different objectives and methodologies, it presented that exploring attentional effort via pupil diameter could be a tool to monitor cognitive load during training sessions and possibly reduce the risk of mental fatigue.

Our findings revealed that players acting in different specific positions (roles) presented distinctive visual behaviours and attentional effort during the Football game. From a practical perspective, coaches and practitioners should consider how best to use and adapt their interventions to improve visual search behaviours and attentional efforts according to their role in the field.

### Limitations

The biggest limitation of this pilot study is the small sample size and the amount of data collected, which do not enable us to demonstrate strongly evidences between the participants. ^31^^,^^32^ Likewise, because we only had three participants, the results could not generalize to the players role but rather to individual variations. It would also be interesting to gather more data in different sporting settings (indoors vs outdoors football training sites) since pupil size diameters could vary depending on the brightness of an environment and coupling decisional actions, as well. ^33^ Additionally, the lack of analysis of motor variables in relation to the visual data limited the possibility of gaining a deeper understanding of perception-action dynamics in a football context. Pupil dilation, reflecting attentional effort, can be a valuable metric to gauge player engagement and fatigue during training sessions and matches. Coaches can use this information to optimise training loads, ensuring players remain focused and attentive throughout.

### Practical applications

***Targeted Skill Enhancement***

Understanding players' visual search rates could help coaches design drills that specifically target areas where players tend to focus less and might achieve better performance. For instance, if a player consistently neglects scanning the space around teammates without the ball, drills could be designed to improve awareness of the movements and positions of the off-the-ball players (teammates and opponents).

Insights into visual search order could guide coaches in developing targeted drills. For example, if a player tends to focus on their opponents before teammates, coaches can design exercises to shift attention priorities. This approach aids in refining decision-making and aligning training with players' natural perceptual tendencies.

***Monitoring Attentional Effort***

Pupil dilation, reflecting attentional effort, could be a valuable tool used to evaluate a player engagement and fatigue during training sessions. Coaches can use this information to optimise training loads, ensuring players remain focused and attentive during specific tactical events.

### Future recommendations

It is proven that conducting pilot studies can assist in assessing whether research is possible on a bigger scale. ^32^ Despite its limitations, the present pilot study gives a new oncoming on-field data that coaches could use to assess and improve their player’s visual behaviour and attentional effort. Building upon these insights, future research should consider expanding the sample size, diversifying participant roles, exploring different environmental settings, and incorporating football specific motor variables into the analysis. These refinements would not only address the current study's constraints but also pave the way for more valid findings.

### Ethics and consent statement

The studies involving human participants were reviewed and approved by the Ethical Committee of Lusófona University (protocol number M25A21) and University College London (protocol number 7067/001). The participants provided their written informed consent to participate in this study, including the data collection and publication.

## Author contributions

FC and CB contributed to the conceptualisation, data collection, data analysis, and writing of the paper. JB contributed to the data analysis and writing of the paper. EH contributed to revising and writing the paper. All authors contributed to the article and approved the submitted version.

## Data Availability

Open Science Framework (OSF): Visual Behaviour and Attentional Effort of Football Players, https://doi.org/10.17605/OSF.IO/WAEYJ. ^30^
- (1) gaze behaviour of football players (Data).csv - (2) Attentional effort of football players (Data).csv Data are available under the terms of the Creative Commons Attribution 4.0 International license (CC-BY 4.0).
