# Supplementary material for: What is the visual behaviour and attentional effort of football players in different positions during a real 11v11 game? A pilot study
Source: F1000Res. 2023 Dec 8;12:679. Originally published 2023 Jun 15. [Version 4] doi: 10.12688/f1000research.134231.4 (PMC10704066; doi:10.12688/f1000research.134231.4)
Supplement: Peer review history

# Reviewer report: Reviewer response for version 4

**DOI:** 10.5256/f1000research.159747.r228718

**Published:** 2023-12-14

**Authors:** Vicente Luis-del Campo

**Affiliations:** 1 Faculty of Sport Sciences, University of Extremadura, Caceres, Spain

I state that I do not have any new comments.

Is the work clearly and accurately presented and does it cite the current literature?

Partly

If applicable, is the statistical analysis and its interpretation appropriate?

Yes

Are all the source data underlying the results available to ensure full reproducibility?

Yes

Is the study design appropriate and is the work technically sound?

Yes

Are the conclusions drawn adequately supported by the results?

Yes

Are sufficient details of methods and analysis provided to allow replication by others?

Partly

Reviewer Expertise:

Visual perception and action in sport

I confirm that I have read this submission and believe that I have an appropriate level of expertise to confirm that it is of an acceptable scientific standard.

# Reviewer report: Reviewer response for version 3

**DOI:** 10.5256/f1000research.159516.r227710

**Published:** 2023-12-07

**Authors:** Vicente Luis-del Campo

**Affiliations:** 1 Faculty of Sport Sciences, University of Extremadura, Caceres, Spain

I would like to congratulate the authors for their efforts in responding to all my requests.

 In my opinion, the article is now in a good shape for indexing due to the well-focused rationale and discussion of the results, and appropiate methodology used to pick up the visual fixations of football players during a specific pre-competitive football game.

 Altogether, we acknowledge authors for this observational pilot experiment, showing also readers an innovative approach to collect the visual activity of football players while performing a training task on the field.

Is the work clearly and accurately presented and does it cite the current literature?

Partly

If applicable, is the statistical analysis and its interpretation appropriate?

Yes

Are all the source data underlying the results available to ensure full reproducibility?

Yes

Is the study design appropriate and is the work technically sound?

Yes

Are the conclusions drawn adequately supported by the results?

Yes

Are sufficient details of methods and analysis provided to allow replication by others?

Partly

Reviewer Expertise:

Visual perception and action in sport

I confirm that I have read this submission and believe that I have an appropriate level of expertise to confirm that it is of an acceptable scientific standard.

# Reviewer report: Reviewer response for version 2

**DOI:** 10.5256/f1000research.154281.r225684

**Published:** 2023-11-27

**Authors:** Guiherme Machado

**Affiliations:** 1 Centre of Research and Studies in Soccer (NUPEF), Universidade Federal de Vicosa, Viçosa, State of Minas Gerais, Brazil

GENERAL COMMENTS

 I would like to congratulate the authors for their effort to advance research regarding this topic. In general, the paper is well structured. I provide some minor comments below.

**Abstract:**

 The abstract provides a clear overview of the study's background, objectives, methods, results, and conclusions.

 Consider mentioning the significance or potential implications of the findings.

**Introduction:**

 The introduction provides a comprehensive overview of the importance of visual perception and attention in football. Consider briefly summarizing previous research on visual perception and attention in different positional roles in football to provide context for the current study by adding a paragraph in this topic (comparison among positions).

**Methods:**

 The methods section adequately describes the participants, equipment, and procedures used.

 Please consider adding a figure showing were the positional roles included in this study usually are positioned and play on the playing field.

**Results:**

 The results section provides descriptive statistics of fixation duration, number of fixations, time to first fixation, and pupil diameter for each player position. It is well written.

**Discussion:**

 The discussion section interpret the findings in light of the research aim and relevant literature.

**Conclusion:**

 The conclusion provides a concise summary ofthe findings.

Is the work clearly and accurately presented and does it cite the current literature?

Yes

If applicable, is the statistical analysis and its interpretation appropriate?

Yes

Are all the source data underlying the results available to ensure full reproducibility?

Yes

Is the study design appropriate and is the work technically sound?

Yes

Are the conclusions drawn adequately supported by the results?

Yes

Are sufficient details of methods and analysis provided to allow replication by others?

Yes

Reviewer Expertise:

Sport psychology, Football, Tactics, Decision-Making, Talent Development & Identification, Performance Analysis.

I confirm that I have read this submission and believe that I have an appropriate level of expertise to confirm that it is of an acceptable scientific standard.

# Response

**Published:** 2023-12-04

**Authors:** Charles Ballet

**Affiliations:** University College London, London, England, UK

We appreciate your thoughful comments and are delighted that you found the paper well-structured. Your feedback is valuable to us. Regarding the minor comments you provided, we have carefully reviewed them and have made the necessary adjustments to enhance the clarity and accuracy of our work. Thank you for taking the time to share your insights, and we look forward to continuously improving our research efforts.

# Reviewer report: Reviewer response for version 2

**DOI:** 10.5256/f1000research.154281.r198481

**Published:** 2023-11-21

**Authors:** Vicente Luis-del Campo

**Affiliations:** 1 Faculty of Sport Sciences, University of Extremadura, Caceres, Spain

In a first round, I suggested authors the introduction of some changes in an attempt to improve the quality of the document. After reading the second version of the authors, I find that some concerns have been correctly addressed. Albeit, other issues remain unclear and/or not addressed. For example:

 - I argued that it was not suitable to include initial hypotheses with exploratory studies, low number of participants and descriptive analyses but the authors still posit these first assumptions for further comparisons between participants in the analysis section. In this line, the authors maintain the presence of hypotheses at the last paragraph of the introduction section. What reasons do the authors handle to maintain these initial assumptions?

 - My two main initial concerns seem not to be fully responded:

 1 - Why did the authors decide to collect and analyze the five seconds before receiving the ball from a teammate on 11v11 games and not 10 seconds as Jordet et al. previously made? I did not find any reason throughout the document justifying this decision. I suggest that the authors could reinforce the idea of analysing the last five seconds of the play sequences because they may be interested in addressing the location of the last fixations performed by the participants just finishing the play sequence.

 2 - Why did the authors not include other motor variables for a better understanding of the perception-action loops experienced by the participants during the experimental task? I did not find any reason throughout the document justifying this decision. The lack of this type of behavioural variables should be stated at least as one limitation of the study or as an improvement for future recommendation section.

 - During the writing of the results, I would recommend authors to concrete what football player achieved the highest or lowest values for fixation durations, number of fixations and time to first fixation at the different AOIs. I also suggested this possible paragraph to enhance the writing of the results: “The RW participant displayed longer fixations on B, ONB and U, compared to the CM and LB participants. The CM participant showed fixations of longer duration on SONB, STB and TB, compared to RW and LB ones. The LB participant made longer fixations only on the TNB, compared to RW and CM ones. For the time to first fixation, the LB participant showed the highest values for the most AOIs, compared to RW and CM ones (e.g., SONB, STNB, STB, TB, U). The RW participant displayed the highest values for B and TNB, compared to CM and LB. Contrary, the CM participant achieved the highest values only for the ONB, compared to RW and LB ones. Finally, to highlight that the CM participant was the footballer who more increased the values for pupil diameter variables from “baseline” to “during play”, compared to the RW and LB participants”. I claimed that this information would help to understand that each participant explored differently the playing field, and what AOIs where more or less relevant for the participants regarding their role in the team. Why did the authors not use this text?

 - I also encouraged authors not to specify the values found in the tables during the wording because they would duplicate information for readers, being redundant during this section. Again, I did not support the use of specific vales for the variables at this discussion section. However, the authors preserve in the use of specific values for the main text and tables, showing them on both occasions. Eliminating this information from the main text, the authors could improve the understanding of the readers about the relevant points/ideas extracted from the data.

 Lastly, I recommended authors to add some practical perspectives for this study. Nevertheless, I did not find any suggestion of the authors at this point. The study has a great practical potential, but the authors should posit some recommendations to orientate training sessions of the coaches.

 I hope that these new comments will serve them in their endeavour to improve the document.

Is the work clearly and accurately presented and does it cite the current literature?

Partly

If applicable, is the statistical analysis and its interpretation appropriate?

Yes

Are all the source data underlying the results available to ensure full reproducibility?

Yes

Is the study design appropriate and is the work technically sound?

Yes

Are the conclusions drawn adequately supported by the results?

Yes

Are sufficient details of methods and analysis provided to allow replication by others?

Partly

Reviewer Expertise:

Visual perception and action in sport

I confirm that I have read this submission and believe that I have an appropriate level of expertise to confirm that it is of an acceptable scientific standard, however I have significant reservations, as outlined above.

# Response

**Published:** 2023-12-04

**Authors:** Charles Ballet

**Affiliations:** University College London, London, England, UK

Reviewer: In a first round, I suggested authors the introduction of some changes in an attempt to improve the quality of the document. After reading the second version of the authors, I find that some concerns have been correctly addressed. Albeit, other issues remain unclear and/or not addressed. For example:

 - I argued that it was not suitable to include initial hypotheses with exploratory studies, low number of participants and descriptive analyses but the authors still posit these first assumptions for further comparisons between participants in the analysis section. In this line, the authors maintain the presence of hypotheses at the last paragraph of the introduction section. What reasons do the authors handle to maintain these initial assumptions?

 Authors: The initial hypotheses have been removed.

 Reviewer: - My two main initial concerns seem not to be fully responded:

 Reviewer: 1 - Why did the authors decide to collect and analyse the five seconds before receiving the ball from a teammate on 11v11 games and not 10 seconds as Jordet et al. previously made? I did not find any reason throughout the document justifying this decision. I suggest that the authors could reinforce the idea of analysing the last five seconds of the play sequences because they may be interested in addressing the location of the last fixations performed by the participants just finishing the play sequence.

 Authors: This was answered in the comments section, It has been now added to the article.

 Reviewer: 2 - Why did the authors not include other motor variables for a better understanding of the perception-action loops experienced by the participants during the experimental task? I did not find any reason throughout the document justifying this decision. The lack of this type of behavioural variables should be stated at least as one limitation of the study or as an improvement for future recommendation section.

 Authors: This has been now included in the limitations of the study.

 Reviewer: - During the writing of the results, I would recommend authors to concrete what football player achieved the highest or lowest values for fixation durations, number of fixations and time to first fixation at the different AOIs. I also suggested this possible paragraph to enhance the writing of the results: “The RW participant displayed longer fixations on B, ONB and U, compared to the CM and LB participants. The CM participant showed fixations of longer duration on SONB, STB and TB, compared to RW and LB ones. The LB participant made longer fixations only on the TNB, compared to RW and CM ones. For the time to first fixation, the LB participant showed the highest values for the most AOIs, compared to RW and CM ones (e.g., SONB, STNB, STB, TB, U). The RW participant displayed the highest values for B and TNB, compared to CM and LB. Contrary, the CM participant achieved the highest values only for the ONB, compared to RW and LB ones. Finally, to highlight that the CM participant was the footballer who more increased the values for pupil diameter variables from “baseline” to “during play”, compared to the RW and LB participants”. I claimed that this information would help to understand that each participant explored differently the playing field, and what AOIs where more or less relevant for the participants regarding their role in the team. Why did the authors not use this text?

 Authors: Done.

 Reviewer: - I also encouraged authors not to specify the values found in the tables during the wording because they would duplicate information for readers, being redundant during this section. Again, I did not support the use of specific vales for the variables at this discussion section. However, the authors preserve in the use of specific values for the main text and tables, showing them on both occasions. Eliminating this information from the main text, the authors could improve the understanding of the readers about the relevant points/ideas extracted from the data.

 Lastly, I recommended authors to add some practical perspectives for this study. Nevertheless, I did not find any suggestion of the authors at this point. The study has a great practical potential, but the authors should posit some recommendations to orientate training sessions of the coaches.

 Authors: Done.

 Reviewer: I hope that these new comments will serve them in their endeavour to improve the document.

 Authors: We highly value the reviewer's feedback and constructive criticism, both of which have significantly contributed to enhancing the overall quality of the article.

# Reviewer report: Reviewer response for version 1

**DOI:** 10.5256/f1000research.147268.r178912

**Published:** 2023-07-17

**Authors:** Vicente Luis-del Campo

**Affiliations:** 1 Faculty of Sport Sciences, University of Extremadura, Caceres, Spain

*Comments to the Authors:

 The submitted manuscript entitled “What is the visual behaviour and attentional effort of football players in different positions during a real 11v11 game?” describes an interesting study about the visual and attentional demands that young athletes in football have when played some specific game sequences in the playing field.

 Firstly, I appreciate your submission on various accounts. To exemplify, it is an innovative study because there are few studies in the literature about visual perception in sport collecting visual fixations of athletes with portable eye trackers in the playing field. Therefore, this study allowed to evaluate the visual behaviours and attention of football players with an in situ approach during real 11 vs 11 sequences of the play. As a result, the ecological validity of this study was guaranteed because authors used representative tasks for the study of visual behaviours in a naturalistic environment. Additionally, I argue that the rationale of the study is well-focused towards the relevance of the perceptual-cognitive skills and visual attention on the athletes´ performance (e.g, anticipation and decision-making). I also agree with the decision of authors of starting the exploration of footballers´ visual and attention activity with a pilot study because this type of studies would: i) provide an initial tendency of the data, ii) enhance a better replication of the same study in further attempts (e.g., improving reliability of research tools and/or designs, etc.), and iii) achieve a higher external validity of the data if a large sample of participants may be recruited.

 Nevertheless, I have two main concerns that require contemplation and appropriate attention in revising the document if it is to contribute appropriately to the extant literature. These two concerns are related to the selected time to analyse fixations and the lack of integration between visual fixations and other behavioural measures of movement:

1. Why did the authors decide to collect and analyze the five seconds before receiving the ball from a teammate on 11v11 games? What is the rationale for this decision? The authors claim that Jordet et al. used a temporal window of 10 seconds for the team possessing the ball, then, why did the authors consider that five seconds would be enough to scan correctly and sufficiently the visual activity of the football players?
2. The existing studies about visual perception in sport have usually used not only visual variables but also other motor outcomes due to the strong relation between perception-action loops. However, there is an absence of measures related to the motor behaviour of athletes in this study. Why did the authors not include this type of behavioural variables when players performed their specific actions on-field? (e.g., decision-making, number of correct passes performed, etc.). The combined analysis of visual information and movement would offer a better understanding of the underlying cognitive processes supporting performance of the players for this tactical sport.

These reservations should be clarified and justified throughout the manuscript before a decision can be made on publication.

**Introduction**

 In my opinion, the background of the paper offers a general viewing of the state-of-the art about visual perception and attention in sport. However, it would be interesting to add more existing studies in football that used variables related to visual performance to address sport performance in controlled laboratory settings. This point would strengthen the need of accumulating more evidence using on-field studies with representative procedures and designs to test visual and motor behaviours of footballers.

 After reading this section, it seems clear that there is a lack of studies assessing visual behaviours in the playing field. For an exception, the authors highlight the observational study conducted by Aksum et al. (2021) with midfield footballers while playing a real 11v11 match. This is a similar study compared to Ballet and colleagues, but these last authors have now included the gaze behaviours of footballers with different roles and visual attention. I would also encourage authors to introduce another recent published paper driven by Luis-del Campo and colleagues (2023) ^1^ because they used ocular metrics and saccadic features as biomarkers of the mental load suffered by football players when performed a training session with manipulation of the available time to complete the goals of the tasks. This study would provide authors some interesting results to compare with your data, for instance, of pupil size.

 Luis-Del Campo, V., Morenas Martín, J., León Llamas, J. L., Ortega Morán, J. F., Díaz-García, J., & García-Calvo, T. (2023). Influence of the time-task constraint on ocular metrics of semi-elite soccer players. *Science & medicine in football*, 1–8. Advance online publication. https://doi.org/10.1080/24733938.2023.2172203

 Additionally, the authors should include other studies that used ocular metrics in footballers. For example, lower pupil diameter was associated with higher values of tactical knowledge (Cardoso et al. 2019) ^2^ and better tactical behaviour efficiency (Cardoso et al. 2021) ^3^.

 I am not sure if it would be appropriate for a pilot study to drive hypotheses with specific predictions about the impact of role position on visual behaviours when the authors show no previous studies for a different visual exploration of the playing field regarding the specific role in the team. Thus, the number of total fixations collected (n=166) seems scarce to conclude these initial assumptions. Indeed, the authors state at the statistical analysis that only descriptive statistics were used. What is the authors´ opinion about this issue?

**Method**

 It would be fine to add more specific information about the visual and motor experiences of footballers (e.g., the average of hours training by week, or the average of hours watching football matches on TV or in the field).

 Again, I find lost a scientific approach to decide that the five seconds before receiving the ball from their teammate were sufficient to fully understand the visual behaviours of the three footballers during 11 vs 11 sequences of play. What do the authors mean when refer to “…interpret another understanding of visual strategies used during a short period of time”? Please, clarify this point.

 Again, the authors did not include any variable related to decisional and/or motor behaviours. The authors should clarify those reasons that prevented them the use of these variables while participants wearing the eye tracking glasses during five minutes of the 11v11 pre-competitive football game. In my opinion, this should be stated as another limitation of the study.  

 I guess that the number of calibration points was 1 when the authors stated “…participants to focus on the center of the calibration card held in front of them for five seconds”, didn't they? If true, they should specify this technical detail.

 The specifications showed by the authors were robust for the identification of fixations and search rate. For the search order of fixations, I would recommend authors to use the Graphos software for a better understanding of this type of analysis of fixations. This software would also help readers to better interpret the relevance of certain AOIs and the order in which the footballers moved their gaze from one AOI to another one.

 The pupil size has showed sensitiveness to different light conditions (Wyatt 1995) ^4^ but also to cognitive states of participants (Beatty, 1982; Mahanama et al., 2022; Mathôt et al., 2015) ^5^^,^^6^^,^^7^. Taking into consideration that the pupil dilation showed responsiveness to different external and/or internal variables, I wonder if the three participants had the same conditions to measure the attentional effort, at the baseline and at a “post-stimulus”, because their visual fixations were collected at different moments of the 20-min 11v11 pre-competitive football game. I would like to know the opinion of the authors about this issue, and I ask them if they consider that the impossibility of measuring simultaneously would be a limitation of the study.

**Statistical analysis and results**

 During the writing of the results, I would add the information about what participant achieved the highest or lowest values for fixation durations, number of fixations and time to first fixation at the different AOIs.

 To exemplify, for the fixation durations, the RW participant displayed longer fixations on B, ONB and U, compared to the CM and LB participants. The CM participant showed fixations of longer duration on SONB, STB and TB, compared to RW and LB ones. The LB participant made longer fixations only on the TNB, compared to RW and CM ones. For the time to first fixation, the LB participant showed the highest values for the most AOIs, compared to RW and CM ones (e.g., SONB, STNB, STB, TB, U). The RW participant displayed the highest values for B and TNB, compared to CM and LB. Contrary, the CM participant achieved the highest values only for the ONB, compared to RW and LB ones. Finally, to highlight that the CM participant was the footballer who more increased the values for pupil diameter variables from “baseline” to “duringplay”, compared to the RW and LB participants.

 This information would help to understand that each participant explored differently the playing field, and what AOIs where more or less relevant for the participants regarding their role in the team. Contrary, I would not specify the values found in the tables during the wording because the authors may duplicate information for readers or be redundant during the presentation of their results.

**Discussion**

 The authors have provided different explanations for their results found, also comparing them to other previous specific studies. I congratulate them because they found well-focused accounts in this attempt.

 Again, the authors should use the results of Luis-del Campo et al. (2023) ^1^ to compare them with those found for their attentional effort variable. This comparison would enrich the discussion section and may show how the pupil diameter shows a similar or different responsiveness regarding the skill level of footballers.

 I would recommend authors more specification when talked about the practical perspective. In this vein, it would be fine that the authors could show some examples of modifications during 11 vs 11 play games to improve visual behaviours of footballers regarding their player role.

*Specific comments to the Authors:

**Introduction**

 The authors refer to decision-making as an “ability”. Are they suggesting that decision-making could not be learned (i.e., natural or innate), or aimed at achieving a goal, as opposed to a “skill”?

 I would recommend authors to delete the last “,” at the next phrase: “….body, and positioning, vary continuously”.

 I would recommend authors to change “when talking about” by “for” at the next phrase: “Therefore, visual perception is crucial when talking about spatial awareness”.

 I would recommend authors to add “up” after the verb “picked”, at the next phrase: “…is mainly picked and processed”.

 I would recommend authors that in this phrase “….can vary depending on the skill level of an athlete”, they would add this: “…..can vary depending on the skill level of an athlete and task constraints existing in the sport environments”.

 I would recommend authors to change “fixate on” by “fixate at”. For example: “Furthermore, midfield players fixate more on the player in possession of the ball during a defensive phase of play than during an attacking phase”. Please, change throughout the document if necessary again.

**Discussion**

 I would not introduce the specific vales for the variables at this discussion section.

 I hope that these comments will serve you well in your efforts to improve your manuscript.

Is the work clearly and accurately presented and does it cite the current literature?

Partly

If applicable, is the statistical analysis and its interpretation appropriate?

Yes

Are all the source data underlying the results available to ensure full reproducibility?

Yes

Is the study design appropriate and is the work technically sound?

Yes

Are the conclusions drawn adequately supported by the results?

Yes

Are sufficient details of methods and analysis provided to allow replication by others?

Partly

Reviewer Expertise:

Visual perception and action in sport

I confirm that I have read this submission and believe that I have an appropriate level of expertise to confirm that it is of an acceptable scientific standard, however I have significant reservations, as outlined above.

## References

[1] : Influence of the time-task constraint on ocular metrics of semi-elite soccer players. *Sci Med Footb* .2023; 10.1080/24733938.2023.2172203 1-8 10.1080/24733938.2023.2172203 36695090

[2] : Young Soccer Players With Higher Tactical Knowledge Display Lower Cognitive Effort. *Percept Mot Skills* .2019;126(3) : 10.1177/0031512519826437 499-514 10.1177/0031512519826437 30744488

[3] : How Does Cognitive Effort Influence the Tactical Behavior of Soccer Players?. *Percept Mot Skills* .2021;128(2) : 10.1177/0031512521991405 851-864 10.1177/0031512521991405 33517852

[4] : The form of the human pupil. *Vision Research* .1995;35(14) : 10.1016/0042-6989(94)00268-Q 2021-2036 10.1016/0042-6989(94)00268-Q 7660606

[5] : Task-evoked pupillary responses, processing load, and the structure of processing resources. *Psychological Bulletin* .1982;91(2) : 10.1037/0033-2909.91.2.276 276-292 10.1037/0033-2909.91.2.276 7071262

[6] : Eye Movement and Pupil Measures: A Review. *Frontiers in Computer Science* .2022;3: 10.3389/fcomp.2021.733531 10.3389/fcomp.2021.733531

[7] : New Light on the Mind's Eye: The Pupillary Light Response as Active Vision. *Curr Dir Psychol Sci* .2015;24(5) : 10.1177/0963721415593725 374-378 10.1177/0963721415593725 26494950 PMC4601080

# Response

**Published:** 2023-08-05

**Authors:** Charles Ballet

**Affiliations:** University College London, London, England, UK

**Reviewer:** The submitted manuscript entitled “What is the visual behaviour and attentional effort of football players in different positions during a real 11v11 game?” describes an interesting study about the visual and attentional demands that young athletes in football have when played some specific game sequences in the playing field.

 Firstly, I appreciate your submission on various accounts. To exemplify, it is an innovative study because there are few studies in the literature about visual perception in sport collecting visual fixations of athletes with portable eye trackers in the playing field. Therefore, this study allowed to evaluate the visual behaviours and attention of football players with an in-situ approach during real 11 vs 11 sequences of the play. As a result, the ecological validity of this study was guaranteed because authors used representative tasks for the study of visual behaviours in a naturalistic environment. Additionally, I argue that the rationale of the study is well-focused towards the relevance of the perceptual-cognitive skills and visual attention on the athletes´ performance (e.g, anticipation and decision-making). I also agree with the decision of authors of starting the exploration of footballers´ visual and attention activity with a pilot study because this type of studies would: i) provide an initial tendency of the data, ii) enhance a better replication of the same study in further attempts (e.g., improving reliability of research tools and/or designs, etc.), and iii) achieve a higher external validity of the data if a large sample of participants may be recruited.

**Authors:** We appreciate your general comments.

**Reviewer:** Why did the authors decide to collect and analyze the five seconds before receiving the ball from a teammate on 11v11 games? What is the rationale for this decision? The authors claim that Jordet et al. used a temporal window of 10 seconds for the team possessing the ball, then, why did the authors consider that five seconds would be enough to scan correctly and sufficiently the visual activity of the football players?

**Authors:** Thank you for your query regarding our decision to collect and analyse data for a 5-second duration before players receive the ball from a teammate in 11v11 games. We understand that the present study is dissimilar to others, especially considering Jordet et al.'s used of a 10-second temporal window for teams possessing the ball in their study. It's also worth pointing out that while they focused on scanning frequency (head movement counts) to explore perceptual outcome measures over a 10-second window, our emphasis was on gaze behaviour (eye movements) which are measured in milliseconds.

 While a 10-second window could potentially offer more comprehensive data, we carefully considered the balance between data richness and practical application. Longer timeframes could lead to more information, but they might also overwhelm coaching staff with a surplus of data to interpret and apply effectively. Moreover, to mimic the actual game, we intended to reduce the available time of the players actions. As this was a pilot study, our goal was to lay the groundwork for future investigations, and we believe that the insights gained from a 5-second window still provide valuable preliminary findings. In future studies, we can explore longer temporal windows to delve deeper into players' visual behaviours and decision-making processes. It could be interesting to investigate whether analysing visual activity within 5 or 10 seconds would be more useful for coaches and stakeholders. Understanding the optimal duration for capturing essential moments during anticipation and decision-making could have practical implications for player development and coaching strategies in tactical sports like football. We will keep this in mind as we continue to expand our research in this area. 

**Reviewer:** Why did the authors not include this type of behavioural variables when players performed their specific actions on-field? (e.g., decision-making, number of correct passes performed, etc.). The combined analysis of visual information and movement would offer a better understanding of the underlying cognitive processes supporting performance of the players for this tactical sport.

**Authors:** We acknowledge the importance of incorporating movement-related measures to gain a deeper understanding of the cognitive processes underlying player performance in football, especially considering the potential differences in visual behaviour and attentional effort based on the various football players' roles and positions. As we move forward with our research, we will certainly include motor behaviour measures in subsequent experiments, allowing us to provide a more comprehensive analysis of the interplay between visual perception and on-field actions. This integration could potentially reveal how visual information processing influences players' actions on the field.

**Introduction**

**Reviewer:** I would also encourage authors to introduce another recent published paper driven by Luis-del Campo and colleagues (2023) ^1^ because they used ocular metrics and saccadic features as biomarkers of the mental load suffered by football players when performed a training session with manipulation of the available time to complete the goals of the tasks. This study would provide authors some interesting results to compare with your data, for instance, of pupil size.

**Authors:** Added.

**Reviewer:** Additionally, the authors should include other studies that used ocular metrics in footballers. For example, lower pupil diameter was associated with higher values of tactical knowledge (Cardoso et al. 2019) ^2^ and better tactical behaviour efficiency (Cardoso et al. 2021) ^3^ .

**Authors:** We have aware of both researches, however the instrument and the software used are quite different from our study. We believe that we need to collect and discuss the data using similar procedures and instruments, since it is our intention to establish a research guide in this scientific domain (improving the reliability of the data). 

**Reviewer:** I am not sure if it would be appropriate for a pilot study to drive hypotheses with specific predictions about the impact of role position on visual behaviours when the authors show no previous studies for a different visual exploration of the playing field regarding the specific role in the team. Thus, the number of total fixations collected (n=166) seems scarce to conclude these initial assumptions. Indeed, the authors state at the statistical analysis that only descriptive statistics were used. What is the authors´ opinion about this issue?

**Authors:** We appreciate your expert opinion, and our future research will confirm or not the differences between specific position (and roles) of football players (different competitive levels).

**Method**

**Reviewer:** It would be fine to add more specific information about the visual and motor experiences of footballers (e.g., the average of hours training by week, or the average of hours watching football matches on TV or in the field).

**Authors:** Added.

**Reviewer:** Again, I find lost a scientific approach to decide that the five seconds before receiving the ball from their teammate were sufficient to fully understand the visual behaviours of the three footballers during 11 vs 11 sequences of play. What do the authors mean when refer to “…interpret another understanding of visual strategies used during a short period of time”? Please, clarify this point.

**Authors:** It is about the representativeness of the actual game. Since the players had less and less time to see and to act, our intention was to provide other visual possibilities.

**Reviewer:** Again, the authors did not include any variable related to decisional and/or motor behaviours. The authors should clarify those reasons that prevented them the use of these variables while participants wearing the eye tracking glasses during five minutes of the 11v11 pre-competitive football game. In my opinion, this should be stated as another limitation of the study.  

**Authors:** Added. 

**Reviewer:** I guess that the number of calibration points was 1 when the authors stated “…participants to focus on the center of the calibration card held in front of them for five seconds”, didn't they? If true, they should specify this technical detail.

**Authors**: We included more information, namely: “To ensure high gaze data quality, calibration procedures were carried out by asking the participants to focus on the center-point of the calibration card held in front of them for five seconds.”

**Reviewer:** The pupil size has showed sensitiveness to different light conditions (Wyatt 1995) ^4^ but also to cognitive states of participants (Beatty, 1982; Mahanama et al., 2022; Mathôt et al., 2015) ^5^ ^,^ ^6^ ^,^ ^7^ . Taking into consideration that the pupil dilation showed responsiveness to different external and/or internal variables, I wonder if the three participants had the same conditions to measure the attentional effort, at the baseline and at a “post-stimulus”, because their visual fixations were collected at different moments of the 20-min 11v11 pre-competitive football game. I would like to know the opinion of the authors about this issue, and I ask them if they consider that the impossibility of measuring simultaneously would be a limitation of the study.

**Authors: **We appreciate your suggestion, however we used the Tobi out-field lenses.

**Statistical analysis and results**

**Reviewer:** During the writing of the results, I would add the information about what participant achieved the highest or lowest values for fixation durations, number of fixations and time to first fixation at the different AOIs.

 To exemplify, for the fixation durations, the RW participant displayed longer fixations on B, ONB and U, compared to the CM and LB participants. The CM participant showed fixations of longer duration on SONB, STB and TB, compared to RW and LB ones. The LB participant made longer fixations only on the TNB, compared to RW and CM ones. For the time to first fixation, the LB participant showed the highest values for the most AOIs, compared to RW and CM ones (e.g., SONB, STNB, STB, TB, U). The RW participant displayed the highest values for B and TNB, compared to CM and LB. Contrary, the CM participant achieved the highest values only for the ONB, compared to RW and LB ones. Finally, to highlight that the CM participant was the footballer who more increased the values for pupil diameter variables from “baseline” to “during play”, compared to the RW and LB participants.

 This information would help to understand that each participant explored differently the playing field, and what AOIs where more or less relevant for the participants regarding their role in the team. Contrary, I would not specify the values found in the tables during the wording because the authors may duplicate information for readers or be redundant during the presentation of their results.

**Authors:** In future research we want to increase the sample size and use comparative methods.

**Discussion**

**Reviewer:** The authors have provided different explanations for their results found, also comparing them to other previous specific studies. I congratulate them because they found well-focused accounts in this attempt.

 Again, the authors should use the results of Luis-del Campo et al. (2023) ^1^ to compare them with those found for their attentional effort variable. This comparison would enrich the discussion section and may show how the pupil diameter shows a similar or different responsiveness regarding the skill level of footballers.

**Authors: **Added.

 “Interestingly, a recent study which looked at evaluating mental load (attentional effort) in training sessions, showed that the pupil diameter of soccer players changes depending on the time spent training ^5^. Although this study had different objectives and methodologies, it presented that exploring attentional effort via pupil diameter could be a tool to monitor cognitive load during training sessions and possibly reduce the risk of mental fatigue.”

*Specific comments to the Authors:

**Introduction**

**Reviewer:** I would recommend authors to delete the last “,” at the next phrase: “….body, and positioning, vary continuously”.

**Authors: **Done.

**Reviewer:** I would recommend authors to change “when talking about” by “for” at the next phrase: “Therefore, visual perception is crucial when talking about spatial awareness”.

**Authors: **Done.

**Reviewer:** I would recommend authors to add “up” after the verb “picked”, at the next phrase: “…is mainly picked and processed”.

**Authors: **Done.

**Reviewer:** I would recommend authors that in this phrase “….can vary depending on the skill level of an athlete”, they would add this: “…..can vary depending on the skill level of an athlete and task constraints existing in the sport environments”.

**Authors: **Done.

**Reviewer:** I would recommend authors to change “fixate on” by “fixate at”. For example: “Furthermore, midfield players fixate more on the player in possession of the ball during a defensive phase of play than during an attacking phase”. Please, change throughout the document if necessary again.

**Authors: **Done.

**Authors: **We deeply appreciate your valuable feedback on our manuscript, and we will certainly take your suggestions on board with our future experiments.
